# RETRACTION: The Impact of Negative Pressure Wound Therapy on Surgical Wound Infection, Hospital Stay and Postoperative Complications after Spinal Surgery: A Meta‐Analysis

**DOI:** 10.1111/iwj.70317

**Published:** 2025-03-06

## Abstract

**RETRACTION:** LuS. , YuanZ. , HeX. , DuZ. , and WangY. , “The Impact of Negative Pressure Wound Therapy on Surgical Wound Infection, Hospital Stay and Postoperative Complications after Spinal Surgery: A Meta‐Analysis,” International Wound Journal 21, no. 1 (2024): e14378, 10.1111/iwj.14378.37697710 PMC10784618

The above article, published online on 11 September 2023, in Wiley Online Library (http://onlinelibrary.wiley.com/), has been retracted by agreement between the journal Editor in Chief, Professor Keith Harding; and John Wiley & Sons Ltd. Following an investigation by the publisher, all parties have concluded that this article was accepted solely on the basis of a compromised peer review process. The editors have therefore decided to retract the article. The authors did not respond to our notice regarding the retraction.

**RETRACTION:** S. Lu , Z. Yuan , X. He , Z. Du , and Y. Wang , “The Impact of Negative Pressure Wound Therapy on Surgical Wound Infection, Hospital Stay and Postoperative Complications after Spinal Surgery: A Meta‐Analysis,” International Wound Journal 21, no. 1 (2024): e14378, 10.1111/iwj.14378.37697710 PMC10784618

The above article, published online on 11 September 2023, in Wiley Online Library (http://onlinelibrary.wiley.com/), has been retracted by agreement between the journal Editor in Chief, Professor Keith Harding; and John Wiley & Sons Ltd. Following an investigation by the publisher, all parties have concluded that this article was accepted solely on the basis of a compromised peer review process. The editors have therefore decided to retract the article. The authors did not respond to our notice regarding the retraction.

